# Comparative evaluation of changes in levels of cytokine – IL-1β in GCF of adult population who have undergone piezocision and corticotomy assisted orthodontics- an in vivo study

**DOI:** 10.1186/s12903-025-06686-9

**Published:** 2025-08-23

**Authors:** Karthika Nambiar, Ranjit Kamble, Srushti Atole, Nandlal Toshniwal, Ruchika Pandey

**Affiliations:** 1 Department of Orthodontics and Dentofacial Orthopedics, Sharad Pawar Dental College, Datta Meghe Institute of Higher Education and Research, Wardha, Maharashtra 442001 India; 2 Department of Orthodontics and Dentofacial Orthopedics, Pravara Institute of Medical Sciences, Loni, Maharashtra India

**Keywords:** Piezocision, Corticotomy, IL-1β, Gingival crevicular fluid, Orthodontic acceleration, Bone remodelling

## Abstract

**Background:**

Accelerating orthodontic treatment is crucial in reducing complications such as root resorption, caries, and oral hygiene compromise, especially in adults. Piezocision and corticotomy are surgical techniques that accelerate tooth movement by causing bone remodelling. This study compares and evaluates changes in Interleukin-1β (IL-1β) levels in gingival crevicular fluid (GCF) following piezocision and corticotomy-facilitated orthodontics in adults.

**Aim:**

To assess and compare IL-1β fluctuations in GCF of adult patients undergoing corticotomy and piezocision-assisted orthodontic treatment.

**Materials & methods:**

This split-mouth study consisted of 16 adult patients (18–25 years) who needed premolar extractions. One half of the maxilla was randomly assigned for piezocision and the other half for corticotomy. GCF samples were taken from maxillary canines at varying time intervals: pre-treatment (T0), before surgery (T1), and 24 h (T2), 7 days (T3), and 21 days (T4) following surgery. IL-1β concentrations were quantified using an ELISA kit, and statistical analysis was done.

**Results:**

IL-1β concentrations were highest at 24 h after surgery (T2) in both groups and decreased steadily by day 21 (T4), but were still elevated above pre-treatment levels. IL-1β concentrations were significantly greater in the piezocision group at all time points after surgery than in the corticotomy group (*p* < 0.001), reflecting a more intense inflammatory reaction and greater bone cell metabolism.

**Conclusion:**

This study suggests that piezocision elicits a more intense biological response than corticotomy, as reflected by higher IL-1β levels in gingival crevicular fluid. While this may indicate a potential for enhanced bone remodelling and accelerated tooth movement, further research is needed to directly correlate cytokine expression with the rate of orthodontic tooth movement.

## Background

Orthodontic treatment in adults presents unique challenges due to a slower metabolic rate and reduced bone turnover compared to adolescents, which often leads to prolonged treatment durations. Extended treatment time increases the risk of complications such as root resorption, dental caries, and compromised oral hygiene maintenance. Therefore, there is a growing interest in developing techniques that can safely accelerate orthodontic tooth movement and reduce overall treatment duration [1, 2]. 

Orthodontic tooth movement is mediated by bone remodelling, which occurs in response to mechanical forces applied to the periodontal ligament and surrounding alveolar bone. This process triggers the release of various biochemical markers, including cytokines like Interleukin-1β (IL-1β), IL-6, and Tumour Necrosis Factor-α (TNF-α), which promote inflammation and osteoclast activity [3, 4]. These molecules are detectable in gingival crevicular fluid (GCF), which serves as a non-invasive diagnostic medium reflecting the underlying biological events associated with orthodontic force application. Because conventional techniques of orthodontics usually render treatment longer than necessary, various newer approaches aim to enhance speed in tooth movement [5]. 

In recent years, Periodontally Accelerated Osteogenic Orthodontics (PAOO) has emerged as a promising approach to reduce treatment time by enhancing bone metabolism through minor surgical procedures [6]. This approach is based on the Regional Acceleratory Phenomenon (RAP), first described by Harold Frost in 1983, which refers to a temporary increase in bone turnover following surgical injury [7]. RAP peaks within 4–8 weeks and can last up to 24 months [8]. Corticotomy and piezocision are two commonly used surgical techniques that leverage RAP to facilitate faster orthodontic tooth movement.

Corticotomy involves selective decortication of the alveolar bone to stimulate bone remodelling. Although effective, it is relatively invasive and may cause patient discomfort [9]. Modified corticotomy techniques have been introduced to reduce surgical trauma while maintaining efficacy [10]. In contrast, piezocision, introduced by Dibart et al., uses ultrasonic microincisions to create localized bone injury with minimal trauma and faster healing. It has gained popularity due to its minimally invasive nature and promising clinical outcomes in both Class II and Class III malocclusions [11]. 

Cytokines, especially IL-1β, play a key role in bone remodelling and are closely associated with the rate of orthodontic tooth movement. Monitoring IL-1β levels in GCF provides valuable insight into the biological response induced by different surgical acceleration methods. This study was therefore designed to evaluate and compare the fluctuations in IL-1β concentrations in GCF of adults undergoing corticotomy- and piezocision-assisted orthodontic treatment.

## Material & methods

This observational study was conducted at the Department of Orthodontics and Dentofacial Orthopaedics, Sharad Pawar Dental College, in collaboration with the Department of Oral and Maxillofacial Surgery and the Central Research Laboratory, DMIMS (DU), Wardha. This study was conducted after approval from the ethical committee of Datta Meghe Institute of Higher Education & Research (DMIHER), Wardha. (reference no- DMIMS(DU)/IEC/2021)

### Sample size calculation

- A pilot study of 8 patients was conducted which was divided into 2 groups:
  - ➢ Group A- site of piezocision
  - ➢ Group B- site of corticotomy
- Formula used is as follows:
  $$n=2\mathrm{sp}^2{\lbrack\mathrm Z1-a/2+\mathrm Z1-\mathrm\beta\rbrack}^2/\mathrm d^2$$

where,

Z1-α/2 = 1.96.

Z1-β = 0.84.

SP = SD1 + SD2/2.

d = mean2- mean1.

From the pilot study, IL1-β levels were calculated

Where, For Group A- mean ± standard deviation was found to be 0.073 ± 0.035.

For Group B- the mean ± standard deviation was found to be 0.112 ± 0.045.

Standard deviation in first group SD1 = 0.035 Standard deviation in second group SD2 = 0.045 SP = 0.035 + 0.045/2 = 0.04.

SP^2^ = 0.0016.

d = mean2-mean1.

d = 0.112 − 0.073 = 0.039.

d^2^ = 0.001521.

*n* = 2(0.0016) [7.84]/0.001521.

= 16.49.

The sample size is 16.

The study included 16 patients aged 18–25 years with healthy gingiva & periodontium who required premolar extractions. Patients with previous orthodontic treatment, systemic diseases, alveolar bone loss, or periodontal disease were excluded. A split-mouth design was used, with one side assigned for piezocision and the other for corticotomy. Randomization was performed using a simple lottery method, where each patient drew a sealed envelope indicating which side of the maxilla (left or right) would receive piezocision or corticotomy. This ensured unbiased assignment of intervention sides. While the surgeon and operator were aware of the allocation, the investigator collecting and analyzing GCF samples was blinded to the intervention to reduce measurement bias.

### GCF collection

**Fig. 1:**
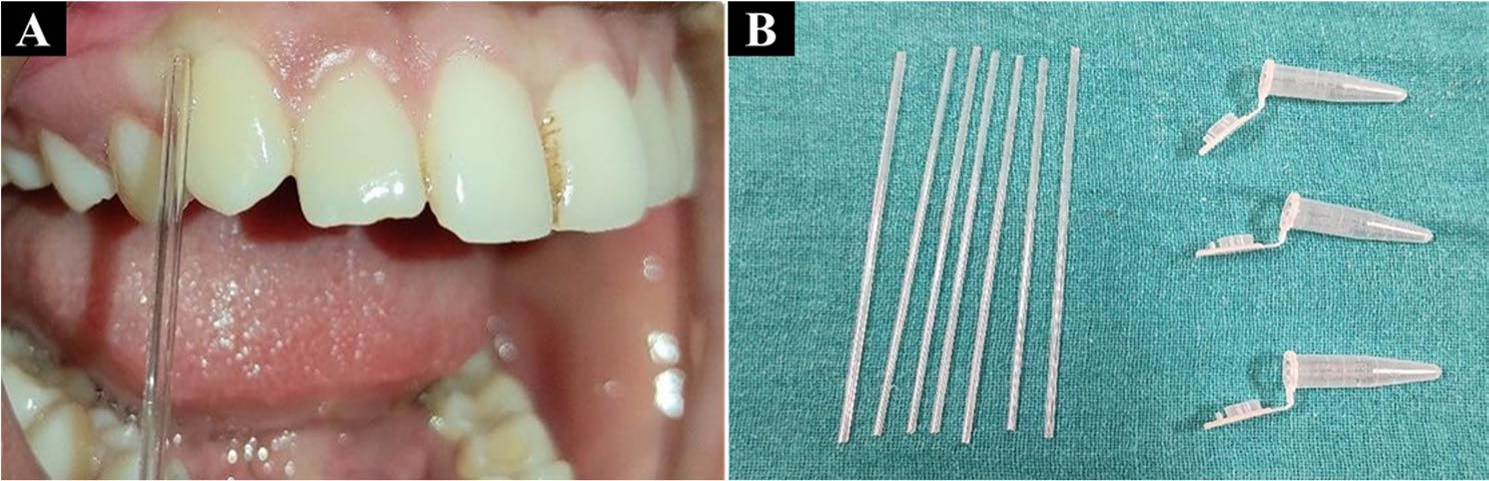
**A** Collection of GCF with Capillary tube, **B **Capillary Tubes & Eppendorf Tubes

Maxillary canines were selected as sampling sites. Supragingival plaque was removed, and the area was air-dried. GCF was collected using capillary tubes inserted into the gingival sulcus for 2–3 min and transferred to Eppendorf tubes (Fig. 1A-B).

Samples were taken at different time intervals:

- T0 – Pre-treatment.
- T1–24 hours post-alignment, before surgery.
- T2–24 hours post-surgery.
- T3–7 days post-surgery.
- T4–21 days post-surgery.

### Levelling and alignment

Orthodontic treatment was initiated using MBT 0.022” brackets with sequential NiTi and stainless-steel (SS) wires. Extractions were performed before placement of 0.019 × 0.025” SS wires. First premolars in all four quadrants were extracted as part of the treatment plan. Extractions were completed before the placement of 0.019 × 0.025” stainless steel wires. Friction mechanics were employed for space closure using active tie-backs, and the force magnitude was standardized to ensure consistency across all subjects. GCF was not collected immediately after extraction to avoid confounding effects, as extraction-induced trauma can trigger a Regional Acceleratory Phenomenon (RAP) and elevate cytokine levels. This would make it difficult to distinguish between biomarker responses due to extractions and those due to the surgical interventions (piezocision and corticotomy). GCF collection commenced 24 hours after placement of the final working archwire, ensuring consistency with the intended time points and minimizing bias.

### Surgical procedure

A single oral and maxillofacial surgeon performed all procedures under local anaesthesia with 2% lignocaine and adrenaline. Patients were rinsed with 2% povidone-iodine pre-surgery, and aseptic protocols were followed. One side of the maxilla was randomly assigned to *corticotomy*, while the other underwent *piezocision*.

**Fig. 2:**
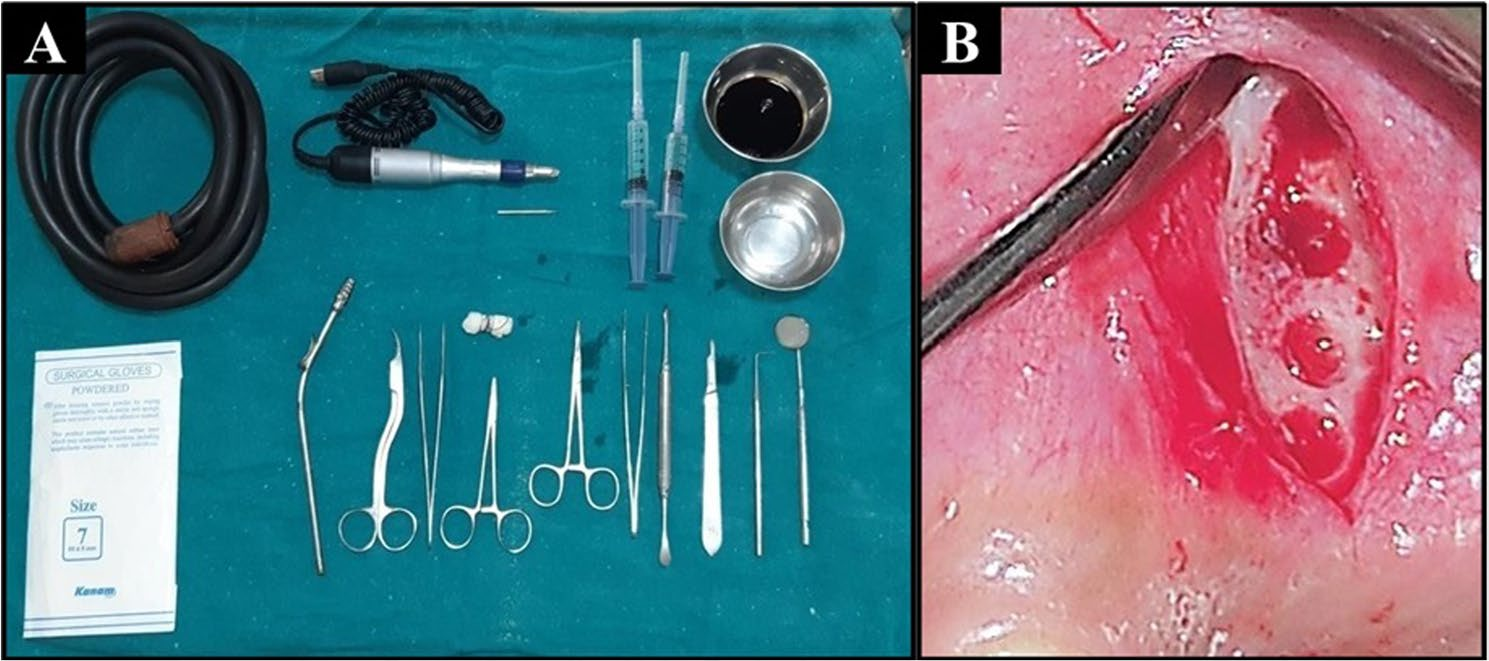
**A** Corticotomy unit, **B** Surgical site

**Fig. 3:**
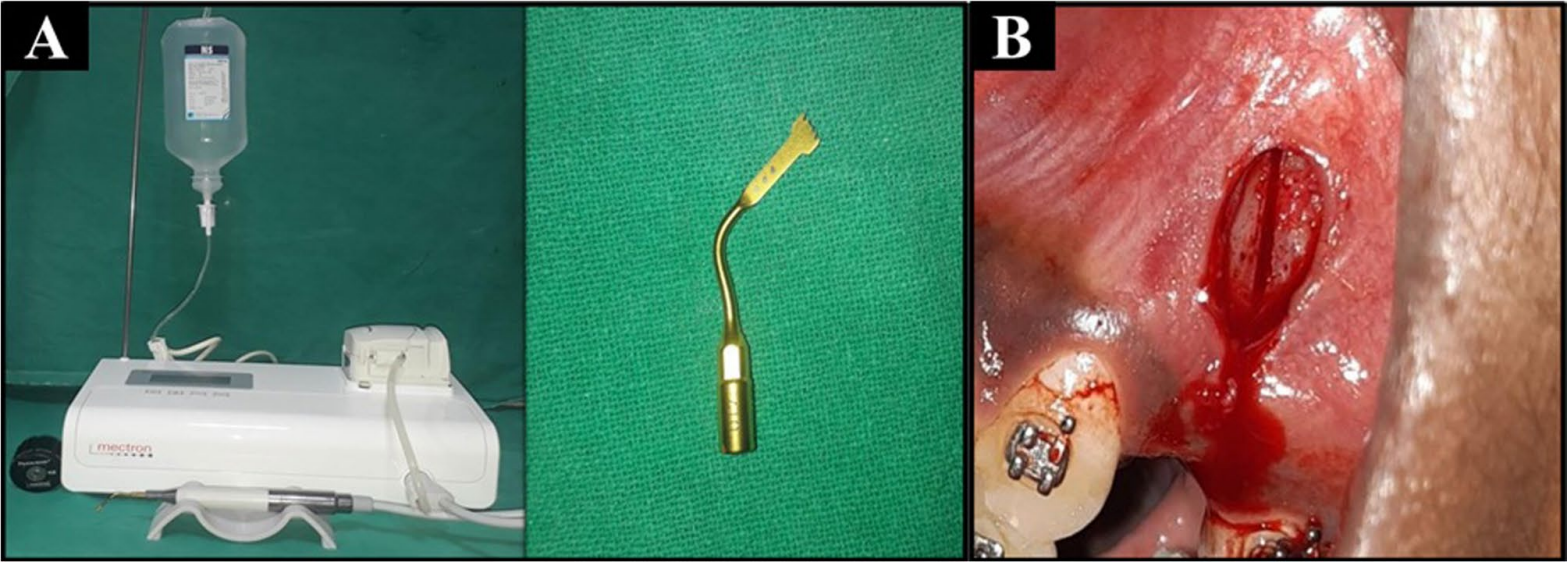
**A **Piezosurgical Unit, **B** Surgical site

- Corticotomy: Vertical bur holes were made in the buccal cortical bone using a carbide round bur (35,000 rpm), penetrating 2 mm into the spongiosa (Fig. 2A-B).

- Piezocision: Microincisions were made using an ultrasonic piezotome − 28–36 kHz frequency (Fig. 3A-B).

Both sites were irrigated with saline and sutured using 4 − 0 silk. GCF samples were collected post-surgery at 24 h, 7 days, and 21 days for biochemical analysis.

### Biochemical analysis

GCF samples were analyzed using a Human IL-1β ELISA kit (Wuhan Fine Biotech Co., Ltd- FineTest) at the Central Clinical Research Lab. Samples were incubated, washed, and processed using a spectrophotometer, generating values for statistical analysis. The process involved preparing reagents, adding samples and biotinylated antibodies to a 96-well plate, incubating for three hours, washing, and adding streptavidin-HRP for further incubation. The reaction was developed with a TMB substrate and stopped with H₂SO₄, and the absorbance was measured using a spectrophotometer. The resulting data were statistically analyzed to compare IL-1β levels between piezocision and corticotomy-assisted orthodontic treatments.

## Statistical analysis

Descriptive and analytical statistics were done. The normality of continuous data was analysed by the Shapiro-Wilk test. As the data followed a normal distribution, parametric tests were used to analyse the data. The independent sample t-test and paired sample t-test were used to check mean differences. The level of significance was kept at *p* < 0.05.[Software: SPSS (Statistical Package for Social Sciences) Version 24.0 (IBM Corporation, Chicago, USA)]

## Results

**Fig. 4:**
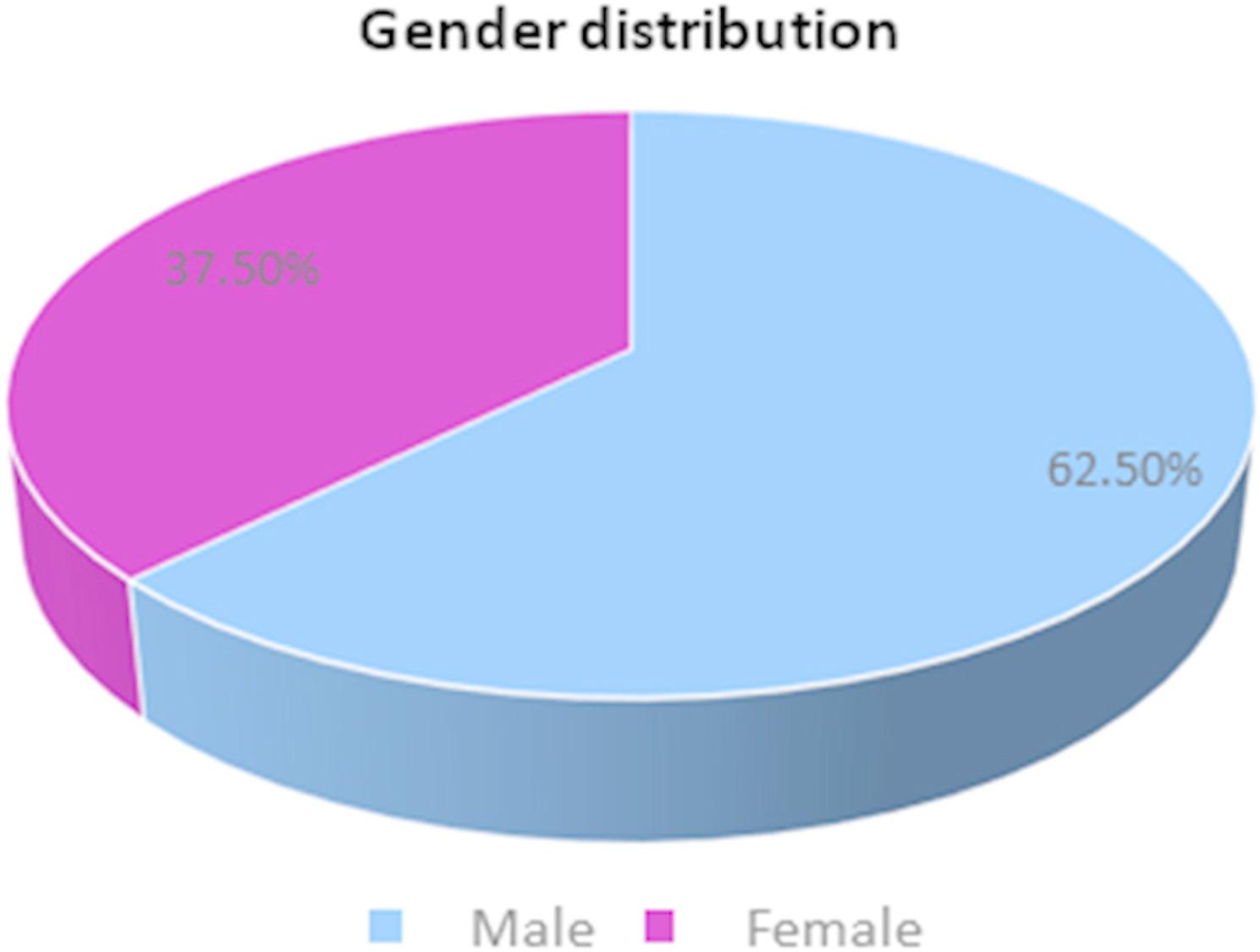
Gender distribution of the study population

This study aimed to evaluate and compare changes in IL-1β concentrations in gingival crevicular fluid (GCF) during corticotomy- and piezocision-assisted orthodontic treatment in adult patients. A total of 16 adult participants (10 males and 6 females) were enrolled in this split-mouth study. Each subject received piezocision on one side of the maxilla and corticotomy on the contralateral side. Demographic details are summarized in Table 1 and Fig. 4.

**Table 1:** Demographic details of the study population

Variables			
Age (*n* = 16)	Mean	S.D.	Range
	20.87	2.41	18–25
Sex	n	%	
Male	10	62.5	
Female	06	37.5

**Fig. 5:**
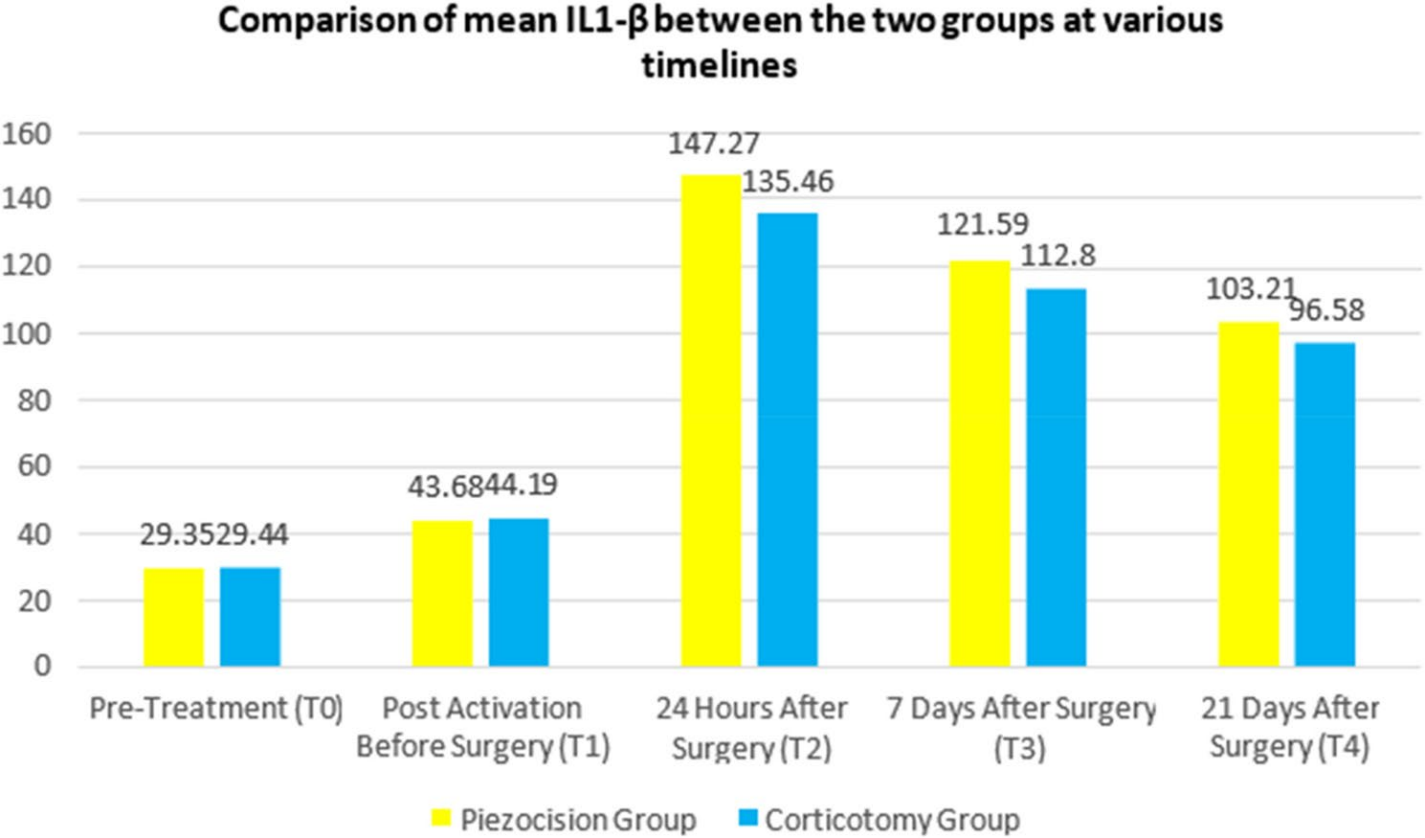
Comparison of mean IL-1β between the two groups at various timelines

The mean IL1-β between the two groups was compared between the two groups at various timelines: (Table 2 & Fig. 5).

**Table 2:** Comparison of mean IL-1β between the two groups at various timelines

Timeline	Groups	*N*	Mean	S.D.	*P*-value^#^
T0	Piezocision	16	29.35	3.66	0.941
	Corticotomy	16	29.44	3.14	
T1	Piezocision	16	43.68	4.62	0.748
	Corticotomy	16	44.19	4.28	
T2	Piezocision	16	147.27	6.33	< 0.001^†^
	Corticotomy	16	135.46	9.85	
T3	Piezocision	16	121.59	5.62	0.002^†^
	Corticotomy	16	112.80	8.73	
T4	Piezocision	16	103.21	6.25	0.005^†^
	Corticotomy	16	96.58	6.12

No statistically significant difference was observed in mean IL-1β levels between the piezocision and corticotomy groups at baseline (T0; *p* = 0.941) or immediately before surgery (T1; *p* = 0.748).

At 24 h post-surgery (T2), IL-1β levels were significantly higher in the piezocision group (mean = 147.27 pg/ml, SD = 6.33) than in the corticotomy group (mean = 135.46 pg/ml, SD = 9.85), with a mean difference of 11.81 pg/ml (95% CI: 7.65 to 15.96; *p* < 0.001). This difference remained statistically significant at 7 days (T3; *p* = 0.002) and 21 days (T4; *p* = 0.005) post-surgery, suggesting a more pronounced inflammatory response following piezocision during the early healing phase.

**Fig. 6:**
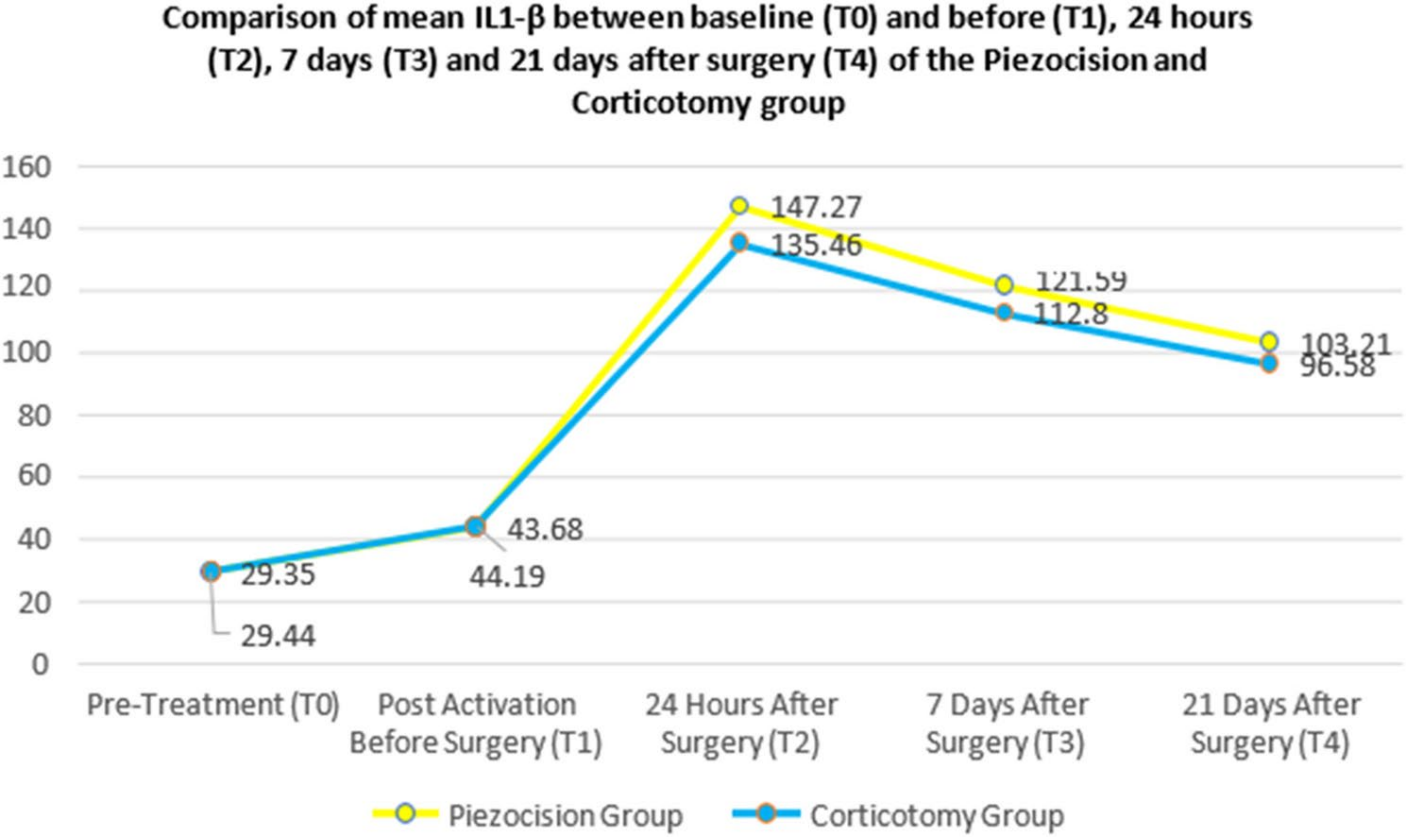
Comparison of mean IL-1β between baseline (T0) and before (T1), 24 hours (T2), 7 days (T3) and 21 days after surgery (T4) of the Piezocision and Corticotomy group

Mean IL-1β concentrations at various time points (T0–T4) within each group are summarized in Table 3 and Fig. 6.

**Table 3:** Comparison of mean IL-1β between baseline and before, 24 h, 7 days and 21 days after surgery (T4) of the piezocision and corticotomy group

Groups	Timeline	*N*	Mean	S.D.	*P*-value
**Piezocision**	T0	16	29.35	3.66	< 0.001
	T1	16	43.68	4.62	
	T0	16	29.35	3.66	< 0.001
	T2	16	147.27	6.33	
	T0	16	29.35	3.66	< 0.001
	T3	16	121.59	5.62	
	T0	16	29.35	3.66	< 0.001
	T4	16	103.21	6.25	
**Corticotomy**	T0	16	29.44	3.14	< 0.001
	T1	16	44.19	4.28	
	T0	16	29.44	3.14	< 0.001
	T2	16	135.46	9.85	
	T0	16	29.44	3.14	< 0.001
	T3	16	112.80	8.73	
	T0	16	29.44	3.14	< 0.001
	T4	16	96.58	6.12

In both the piezocision and corticotomy groups, IL-1β levels increased significantly from baseline (T0) at all subsequent time points (*p* < 0.001), indicating a robust inflammatory and bone remodelling response to orthodontic and surgical interventions.

In the piezocision group, IL-1β levels rose from 29.35 pg/ml at T0 to 43.68 pg/ml at T1, peaking at 147.27 pg/ml at 24 h post-surgery (T2). This was followed by a gradual decline to 121.59 pg/ml at day 7 (T3) and 103.21 pg/ml at day 21 (T4), although levels remained elevated compared to baseline.

Similarly, the corticotomy group demonstrated an increase from 29.44 pg/ml (T0) to 44.19 pg/ml (T1), peaking at 135.46 pg/ml at T2, then declining to 112.80 pg/ml (T3) and 96.58 pg/ml (T4).

Both groups followed a comparable trend, with IL-1β levels rising sharply post-surgery and gradually declining over time. However, the piezocision group consistently exhibited higher cytokine levels at each time point, reflecting a more pronounced biological response.

## Discussion

Orthodontic treatment relies on mechanical forces to induce cellular responses that drive bone remodelling and tooth movement. However, treatment durations often extend over two to three years, particularly in adults, increasing the risk of complications such as root resorption, decalcification, and patient non-compliance [12]. In this context, surgically assisted techniques like corticotomy and piezocision have emerged as effective strategies to accelerate orthodontic tooth movement.

Corticotomy, as demonstrated by Hoogeveen et al. [13] expedites tooth movement by inducing localized bone injury, thereby stimulating the Regional Acceleratory Phenomenon (RAP)—a transient burst in bone turnover and remodelling activity following surgical trauma. In this study, a modified corticotomy approach was employed, limiting the intervention to buccal perforations as described by Suryawanshi et al. [14]. to minimize patient discomfort while retaining clinical efficacy.

Piezocision, introduced by Dibart et al. [15]is a minimally invasive alternative involving ultrasonic micro-incisions that also leverage RAP. Unlike corticotomy, it avoids flap elevation and results in quicker healing, less postoperative morbidity, and better patient acceptance. The present study demonstrates that both techniques activate a significant inflammatory response, as evidenced by elevated levels of Interleukin-1β (IL-1β) in gingival crevicular fluid (GCF), a key biomarker of osteoclastic activity and bone remodelling. Studies by Uematsu et al. [16] and Grieve et al. [17] confirmed that IL-1β levels significantly rise during orthodontic treatment, correlating with tooth movement.

Our findings show that IL-1β levels peaked at 24 h post-surgery (T2) in both groups, with significantly higher concentrations in the piezocision group. This suggests that, despite being less invasive, piezocision may provoke a more robust or prolonged biological response—possibly due to the focused nature of bone trauma from piezotome micro-incisions, which may more effectively stimulate RAP without disrupting surrounding tissues.

Interestingly, the difference in IL-1β levels between the two groups widened progressively after T2, with the piezocision group maintaining higher levels at days 7 (T3) and 21 (T4). This sustained elevation may reflect greater and longer-lasting activation of the inflammatory cascade, resulting in a more efficient bone remodelling environment conducive to accelerated tooth movement. The minimal trauma and continuous vibration generated by piezocision might contribute to prolonged cytokine stimulation compared to the more diffused response from corticotomy.

An important consideration in interpreting these findings is the timing of extractions, which were performed prior to the placement of 0.019” × 0.025” stainless steel wires. GCF sampling did not occur immediately after extraction to avoid confounding IL-1β elevations due to extraction-induced trauma. This precaution ensured that any observed changes in cytokine levels could be attributed to the surgical interventions rather than extraction-related inflammation.

The study design also ensured consistency in mechanics and force application. A split-mouth approach controlled for inter-individual variability, while friction mechanics with active tie-backs minimized biomechanical inconsistencies. This method, supported by Kuhlberg et al. [18] helps optimize space closure. NiTi-closed coil springs delivering a constant 150 g force were used for canine distalization, a method validated by Dixon et al. [19] who observed an average space closure of 0.81 mm per month.

These results are consistent with previous studies. Simre et al. [20] and Alfawal et al. [21] reported that piezocision led to more efficient tooth movement compared to corticotomy. Although Abbas et al. [22] observed greater canine retraction with corticotomy; their study involved conventional (not modified) corticotomy. The higher IL-1β levels observed with piezocision in our study support findings by Rehab Khalil et al. [23] that more pronounced inflammatory responses correlate with faster movement rates.

This study contributes to the understanding of biological mechanisms in surgically accelerated orthodontics. The prolonged IL-1β elevation seen in the piezocision group supports its clinical utility as a minimally invasive yet biologically effective technique. These findings reinforce the clinical potential of piezocision as a minimally invasive and biologically effective technique for accelerating orthodontic treatment in adults. Further studies with larger sample sizes and long-term follow-up are warranted to validate these findings and explore patient-centred outcomes.

## Conclusions

This study showed that both piezocision and corticotomy resulted in a significant increase in IL-1β levels in gingival crevicular fluid, reflecting an active inflammatory and bone remodelling response. Peak levels were seen at 24 h post-surgery and remained elevated for 21 days. The piezocision group consistently exhibited higher IL-1β levels, suggesting a more pronounced biological response. However, as actual tooth movement was not measured, these findings cannot confirm any treatment acceleration. The elevated cytokine levels should be interpreted cautiously and regarded only as indicators of biological activity. Further studies with larger samples and clinical tooth movement data are needed to determine whether this enhanced response translates to improved orthodontic treatment efficiency.

### Limitations

This study has several limitations that must be considered. The sample size was small (*n* = 16), which limits statistical power and generalizability. The 21-day observation period only captures early inflammatory responses and does not reflect long-term biological or clinical outcomes. Importantly, the study did not assess actual orthodontic tooth movement, which limits the ability to draw conclusions about treatment acceleration or efficacy. Furthermore, only healthy young adults were included, excluding patients with systemic conditions or complex skeletal patterns, thereby restricting broader applicability. Operator blinding was not possible due to the surgical nature of interventions, which could introduce bias. Despite these limitations, the study offers valuable preliminary insights into the biological effects of piezocision and corticotomy in orthodontics.

## Data Availability

All data or materials generated or analyzed during this study are included in this article.
